# British Asylums for the Insane

**Published:** 1853-10-01

**Authors:** 


### Art. V.—BRITISH ASYLUMS FOR THE INSANE.

In conformity with our usual custom we now proceed to submit to the consideration of readers an analysis of the Reports forwarded to us by different medical superintendents of the British County Lunatic Asylums, for 1852-3.

We commence with the last annual Report of the *Devon County Lunatic Asylum*, of which Dr. Bucknill is the medical superintendent. According to that physician, 116 patients have been admitted during the year 1852, of whom 51 were males, and 65 females; whilst the total number resident, at the date of his report, was 459 lunatics, 198 being male, and 261 female, patients; thus showing a considerable predominance of the latter sex. During the period above mentioned, 52 inmates were discharged recovered, whilst 30 died, 14 being males, and 16 females, which indicates the recent mortality was low, having only reached 6·6 per cent. of the average number resident, and 5·5 per cent. of the total lunatics under treatment. Respecting some special features noticed at this asylum, we quote the following remarks from Dr. Bucknill's rather brief statement:—

"Three only of the deaths occurred in patients whose disorder was in any degree recent; of these, No. 1034, a female patient, aged 53, had laboured for two months under the delusion that all her food was poisoned, and had consequently refrained from taking nourishment until, when admitted, she was carried to the wards in a state of syncope, from inanition: she was kept alive thirteen days, and then sank from failure of the powers of life. The mucous membrane of the stomach was in a softened state; perhaps the condition of this organ gave origin to the mental delusions. No. 1032, a male patient, was admitted in the last stage of pulmonary consumption, of which he died in twenty-six days. No. 1038, a male patient, aged 66, was admitted with maniacal excitement, and inflammation of the pleura on both sides of the chest, of which he died, forty-six days after admission.

"The remaining twenty-seven cases were all chronic; and the average duration of the residence in the asylum considerably exceeded two years and a half.

"Of the patients who died, five were above 70 years old, five were above 60, eight were above 50, five above forty, four above 30, and three between 20 and 30; the average age of the whole number being 50 years.

"During the past year only five deaths have occurred from general paralysis, being a great decrease from the number occasioned by this fatal disorder during the previous year, when thirteen deaths were caused by it. The average mortality in any particular year appears to depend in no inconsiderable degree upon the number of patients under treatment with this invariably fatal form of disease.

"The health of the patients generally has been very good during the year; and there has been no accident of a serious kind. The immunity we have enjoyed from suicides for several years past must be looked upon rather as a fortunate occurrence than as a result capable of being attained by any amount of precaution."

In reference to occupying the insane residents, this document farther observes:—

"Much attention has been paid to develop the industrial capabilities of the institution. The whole of the clothing, both for the males and females, has for some time past been made at home; and with the exception of one paid shoemaker, the whole of this work has been done by the patients, directed by the ward attendants. A considerable quantity of excellent bedsteads and ward furniture has also been made by the patients; and a brisk manufacture of coir mats, for home use and for sale, is carried on in the wards. No employment, however, is so beneficial, either in an economical or sanitary point of view, as gardening, or spade husbandry; and on this account the limited quantity of ground attached to the institution is a constant source of regret."

Dr. Bucknill subsequently states, regarding mental instruction, which often proves highly useful even amongst insane persons—

"The evening school classes proceed in a satisfactory manner;—the reading classes assist in relieving the monotony and tedium of confinement, and tend to develop in many patients those mental faculties which would otherwise become enfeebled for want of exercise. Attempts are being made, not without a prospect of success, to teach a class of idiots to read; but most of the idiots sent from union houses to this asylum are too old to learn, except in the most imperfect and laborious manner, They are often much older than their appearance would indicate, the childish smallness and rotundity of feature remaining until they are far advanced into adult life. One little woman, with a skull no larger than a cocoa-nut, appears about 16 years old, whereas she is, in fact, 46; and another, who might pass for a child of 9 or 10, is 24 years old. These patients are usually sent to us either because their strength and unrestrained passions make them dangerous inmates of union houses, or because increasing decrepitude, uncleanly habits, and epileptic fits render them extremely helpless, and demand more systematic nursing and attendance than can be procured for them elsewhere. As a general rule, young, docile, and healthy idiots are not sent to the asylum. A considerable amount of improvement and of education, in the wide sense of that term, has been, however, attained in the most unfavourable cases. Strong and healthy, but dangerous, idiots are employed in garden work or domestic occupations; and by avoiding irritation, and exercising perpetual control, they, for the most part, become obedient and docile. By attention to the wants and by reforming the habits of the dirty and helpless they become cleanly, and acquire some degree of self-respect. The wet beds in the idiot wards, containing 64 patients, have been reduced from a nightly average of 40 to an average of less than 4, and these mostly owing to epileptic fits. Some cases have occurred in which idiots who have always lain in loose straw have been uniformly dirty and destructive to clothing, and appeared sunk in the most degraded and hopeless condition, have been raised to some degree of activity, have become cleanly and careful about their own dress, and useful to the ward attendants in dressing other patients, in cleaning floors, crockery, and other simple occupations. More than this could, no doubt, be effected, even with the unpromising cases to be found in our wards, if several skilful instructors were provided, and all the appliances of an idiot school were brought to bear. The soil, however, is sterile; and, under the most diligent cultivation, would, I fear, yield but a scanty return, in proportion to the labour and expense bestowed on it."

Several instructive tables are appended to this report, from which it appears that, amongst the 116 new patients admitted last year, 40 laboured under melancholia, 22 under chronic mania, 15 were affected with recent mania, and 13 with dementia; 7 were epileptics, 6 had symptoms of general paralysis, and 6 were imbeciles or idiots; whilst 4 cases are classed as examples of recurrent mania, 2 of puerperal insanity, and, lastly, 1 case of monomania. Respecting the assigned causes, drunkenness appears to have been the most frequent; 15 instances of this kind, or nearly one-eighth of the whole number, being so reported, which seems to imply that, even in this cider-drinking county, intemperate habits are by no means uncommon. Religious excitement produced mental derangement in 10 patients; 8 became insane through congenital defect; 6 in consequence of pecuniary difficulties; 5 by epilepsy; and 4 from disappointed affections, besides other influences of a special kind, which it is unnecessary now to enumerate. An obituary is added, giving the sex, age, and time of residence in the asylum, of the 30 patients who died. Along with the above particulars, the apparent cause of death, capacity of the cranial cavity, weight of brain, and specific gravity of cerebrum, as also of the cerebellum, are minutely mentioned. These tables are all valuable, and will repay perusal; indeed, statements of the above description cannot but prove of service; and we regret Dr. Bucknill has not extended their number, seeing an institution which annually contains upwards of 450 lunatics, must have afforded ample scope for compiling highly important additional statistical information.

We now proceed to the *Somerset County Asylum*, of which Dr. Boyd is the resident medical superintendent. At this public institution, the number of admissions, during 1852, was 128; of these, 62 were male and 66 female patients; 35 males and 23 females died, whilst 28 of the former, and 40 of the latter sex, were discharged, leaving 342 lunatics in the asylum at the end of the year, of whom 155 were male and 187 female inmates; thus showing that here also, as in the Devon Insane Establishment, mental disease predominated amongst females.

Respecting the mortality, casualties, and accidents, which characterized the past year, Dr. Boyd observes:—

"The mortality amongst the males has been greater than in any previous year; which appears to have been owing to the feeble and hopeless state in which a great number were admitted. Nearly one half of the deaths were from among the admissions of the year; and there are still a great number of infirm cases remaining.

"Coroners' inquests were held in four cases of sudden death, three of which were epileptics; and one a case of fracture of the leg, in which death occurred from inflammation of the lungs twelve days after the accident.

"Five accidents occasioning fractured bones happened during the year, and four of them in the course of a few weeks. The first was that of a male patient, who had been at work whitewashing, and got out and attempted suicide by throwing himself before a wagon, containing four tons of coal, which was passing on the road. The front wheel passed over him, breaking all the ribs and the collar bone on the left side; the lung also was wounded, as air could be felt beneath the skin externally; he was cold, and when placed in bed the pulse was scarcely perceptible. But no unfavourable symptom afterwards appeared; his recovery proceeded rapidly, and at the end of a few weeks he was able to resume his work as a mason. This man is a determined suicide, as he has lately attempted to force his way into the boiler in the wash-house, in presence of the laundress and several patients.

"The second was the case of a female whose arm was fractured from being thrown from her seat by a violent push from another patient; the bone united, and in a short time she recovered the use of her arm.

"The third was the case of a woman, aged 70, who had been recently admitted, and who was also pushed down by a violent patient, and had her arm broken. From her age and infirm state her recovery was much slower than the others.

"The fourth was that of a male patient who had a part of one of his fingers nearly severed, in feeding the chaff cutter, an employment at which he had been injudiciously placed; the wound soon united.

"The fifth was the case of a man of 58 years of age, who had his leg fractured by the overturning of a ladder, and a fall of not more than three feet. He was in indifferent health; the fracture gave him but little pain; on the ninth day after the accident his lungs became affected, and in three days he died from congestion and inflammation of their lower lobes. No case of fracture had occurred in any previous year."

After alluding to various interesting points connected with the proper administration of an asylum for the insane, the reporter next proceeds to discuss the question of medical treatment. Upon this point we cannot do better than quote Dr. Boyd's remarks *in extenso*. When speaking of particular diseases and their management, he says:—

"In epilepsy the tincture of sumbul still seems to mitigate the severity of the fits. In some cases attended with twitchings of the muscles of the face and neck, a solution of atropine applied endermically, after a blister on the front of the neck, has lessened the number of the fits; the strength of the solution was four grains to one ounce of distilled water. Attention has been paid to keeping the bowels open by medicine when the fits are likely to recur, and also to raising the heads of those patients who are subject to fits at night. The relative frequency of fits in the male and female patients, and also during the day and night, may be seen by referring to Table 3 annexed to this Report. Five male and seven female patients have been admitted, and three male and five female epileptic patients have died during the year.

"In the previous reports I have mentioned that the fatal cases in which *general paralysis* was the diagnostic symptom were found, on examination after death, to be accompanied by disease of the spinal cord, the result of inflammation, in which the ventricles and membranes at the base of the brain were generally implicated. Further experience corroborates this statement; and it has rarely happened that there could not be detected a sufficient amount of disease in the spinal cord or base of the brain to lead to the fair presumption that the symptoms were dependent on this cause. In addition to the evidence afforded by a post-mortem examination, a portion of the diseased parts was, in most instances, subjected to a microscopical examination by my experienced friend Mr. Gulliver, who found that the 'exudation corpuscles' were most frequently present in the spinal cord itself, and were similar to those delineated and described by Dr. Bennett in his paper on inflammation of the nervous centres. In the treatment of such cases, attention has been mainly directed to checking the inflammation, with which view the *Liquor Hydrargyri bichloridi* has been given to eight male patients, two of whom are better now than they have been; and one who was confined for several weeks to the web bed, with sores and in a very helpless state, is now able to sit in a chair, and to feed himself, which at one time he was quite unable to do. Another, who was of dirty habits, after taking this medicine for some time, became cleanly, and gained in weight 21 lbs. in six months: two of the cases are gradually becoming worse, and four appear to be stationary.

"Two male patients in a state of raving madness, destructive in their habits, and upon whom medicine had no good effect, derived benefit from being frequently placed in a warm bath for several hours, and the application of cold occasionally at the same time to the head. Dr. Junot's very effective instrument for dry cupping had been tried on a lower extremity of one of those patients, and only quieted him for a short time. Two males and one female had serious tumours of their ears, appearing like wind galls, which afterwards discharged a glairy fluid, accompanied in two of them with slight ulceration.

"The average weight of the brain has this year been $47\frac{1}{3}$ ozs. in the males, and 42 in the females, which in the males only exceeds the average weight of the brain in the sane, which was given in former reports.

"The insane are as subject as others to the ordinary diseases, while in them, for obvious reasons, they are more difficult to detect; and the practitioner will find an intimate knowledge of the general characteristics of disease peculiarly requisite in an asylum, where he has, in most instances, to form his diagnosis without the assistance of his patient. Fatal cases of inflammation either in the chest or abdomen often occur, of which there is no suspicion until, perhaps, a few hours (10 or 12) before death, and in which the precise nature of the disease is only ascertained by dissection. More than half of those who died were found to have had disease of the lungs, most commonly in an acute form with low symptoms, and the immediate cause of death. In about one-fifth of those who died there was disease in the abdomen, chiefly from inflammation and ulceration of the stomach and intestines, affecting the mucous tissues chiefly. Other incidental cases, not of a fatal nature, have occurred, some of which may here be shortly mentioned as having been benefited by certain treatment.

"A male patient in a state of dementia or incoherence, who is in the habit of working at his trade as a painter, was easily affected by the carbonate of lead used in painting, which often produced colic, attended with constipation of the bowels, loss of appetite, and general debility.

The usual remedies in such cases were administered and always found sufficient to relieve the symptoms. He was for some time after his last attack, which occurred eighteen months ago, kept on the use of the pyroligneous acid, in half drachm doses, diluted with water, twice a day, which has been quite effectual as a prophylactic; it has been discontinued for the last seven months, and he has followed his trade without since suffering in the slightest degree. Two cases in young persons of paralysis from lead, rapidly recovered under the use of this remedy, and one old case was benefited. The salutary action of the acetic acid is explained on chemical principles; it is supposed to convert the insoluble and poisonous carbonate of lead into the soluble and comparatively harmless acetate of lead.

"In several obstinate cases of rheumatism, with redness and swelling of the joints, about half an ounce of nitrate of potash in powder, on a piece of *spongio-piline* moistened with warm water, after Dr. Basham's formula, and applied round the part, was found, after a short time, to afford relief. The efficacy of this kind of remedy in subduing inflammation may be ascribed, according to Mr. Gulliver, to the effect of alkaline and earthy neutral salts in thinning the blood and keeping asunder the red corpuscles, so as to prevent their accumulation in, and obstruction of, the minute vessels. Saline purgatives were also given when required; and in some cases a solution of fifteen grains of citric acid and four of hydriodate of potash two or three times a day.

"In the case of a male patient in a state of melancholia, and suffering from tapeworm, one dose of the *Kousso*, or *Bayera anthelmintica*, was found effectual in expelling the worm, although the head was not attached to the expelled part. This man is improved in the state of his mind."

Several instructive tables are appended, besides an obituary, which gives in detail, yet succinctly, an account of the previous condition of the patients who died during the past year, along with the appearances noticed after death, as also the weight of the principal organs. Dr. Boyd being already so well known to the profession for extensive pathological knowledge, it is almost superfluous to say, in now taking leave of that physician and his recent report, that this portion especially merits careful study on the part of every zealous student of psychological medicine.

Unlike the institution which has just occupied our attention, the Report of the *Gloucester County Asylum* does not contain a single observation of any kind, either from the physician-superintendent, Dr. Williams, or Mr. Allen, assistant medical officer. Why this anomaly exists, it is difficult to explain; and we cannot help thinking such an unusual defect as total silence, by the medical attendants of this public establishment for the insane, is far from being desirable, and ought in future to be avoided.

However, the visiting magistrates have prefixed a short annual report to some useful tables drawn up by Dr. Williams, which are worthy of perusal, especially No. 12, wherein various interesting points connected with the mortality recorded are minutely detailed. The facts here described show that the medical officers have paid great attention to pathology; and from this specimen we augur, with some confidence, that any practical remarks from the same source would be equally valuable to, and doubtless favourably received, by the profession. Owing to the unaccountable omission now mentioned, we can therefore only refer to the meagre official statement made by Mr. Hayward, chairman of the visiting magistrates, who states that—

"The course of treatment pursued towards the patients, both mentally and bodily, has tended much to their comfort and general amelioration; and has been so far successful, as to cure, that the Tables show that while the admissions during the year have been 128, the number discharged as cured have been 66. At the close of the last year there only remained in the house, out of 300 patients, 31 who were deemed to be curable, and there are now 26; so that the cures may be considered as exceeding 50 per cent. on the admissions of the year. The general health of the patients has been most satisfactory, and there has been an entire absence of any epidemic during the year: the mortality, however, has been unusually great for this institution, amounting to 45, a number considerably exceeding that of last year, and very much larger than that of former years. The visitors have no means of accounting for this increase, except that, owing to the extraordinarily small mortality of the two or three years preceding 1850, a number of shattered constitutions had outlived the previous years to swell the mortality of 1850 and 1851, and also the circumstances referred to specially in their last report, namely, the very aged and feeble state in which many cases are received into the asylum. During the past year five patients died within three weeks of admission, one of whom was upwards of eighty years of age, two more upwards of seventy, and others above sixty. The visitors think it necessary to refer more particularly to one death, which occurred in the institution in September last, under the following melancholy circumstances. A gentleman who had been an inmate of the asylum many years, usually of quiet habits, but subject to occasional fits of excitement, who had retired to rest tranquilly and in his ordinary health, appeared to have risen from his bed in the night and made an attack upon his window, which he succeeded in breaking through, and precipitating himself to the ground, a distance of more than forty feet, by which his death was caused. The patient had never shown any suicidal propensity, and it would seem very doubtful whether he had any idea of the kind in this instance, or was conscious of the act he was committing. These window frames, attached to the bed-rooms of the opulent patients, had been in use since the opening of the asylum, nearly thirty years ago, and nothing had occurred before to make them appear insecure. An immediate order was made for removing the wooden frames and replacing them by wrought iron of the same pattern. The visitors have the satisfaction in stating, in support of the system of the absolute abolition of every species of mechanical restraint, which has been adopted now for many years in this asylum, that before this unhappy occurrence only one case of suicide had occurred here for upwards of five years and a half, and that the unhappy event above mentioned can in no way be connected with such a cause, as under no circumstances would this unfortunate gentleman have been considered a subject for restraint."

These observations are so far satisfactory, indeed, we wish they had been extended; and whenever subsequent reports appear, we trust the physician-superintendent will not fail to add his quota of professional information.

Although the recent Report of the *Bedford Asylum* contains, as an appendix, several succinct remarks made by the visiting surgeon and superintendent, Mr. Harris, as also the resident medical officer, Mr. Matthews, this official document is so limited in extent, as only to occupy a little more than two pages. Hence, our notice must be brief, and confined to the following extracts, as they will convey some general notion of the chief features characterizing this public establishment.

"The daily average number of patients in the asylum has been 275; for a considerable period there were more than 280; the total number in the asylum during the year, 327.

"At one time there were 284, being 14 more than the asylum could properly accommodate.

"There have been 39 patients discharged, 13 recovered, 11 removed by their friends, improved, and 15 to other asylums.

"There have been 19 deaths, 10 male and 9 female; the causes of death have been various, as stated in the annexed table.

"The mortality during the year has been smaller than it has been for some years, and this irrespective of the small number of admissions.

"The health of the patients has been very good, and no serious accident has occurred.

"We have to report that on the 10th of January of the present year, an unfortunate occurrence took place. A patient named William Dubberly, *alias* Smith, whilst employed in the garden, ran away and made to the river (a short distance from the asylum), into which he deliberately walked and swam off, inviting those who endeavoured to overtake him, to follow; he swam a considerable distance down the stream, and then floated on his back apparently much at his ease; he then turned over and swam a short distance, when he suddenly sank to rise no more."

Notwithstanding physical restraint as a remedial means in the treatment of insanity is now almost universally repudiated throughout the public lunatic asylums of Great Britain, the following paragraph would indicate the Bedford institution to be an exception, seeing the medical officers state their deliberate conviction that—"On the subject of restraint we are still of opinion that in some cases mild mechanical restraint is advisable and beneficial."

Such an admission should not be passed over in silence, and we now draw attention to the point, as it expresses an opinion respecting which many will demur, although conscientiously arrived at by these gentlemen.

The *General Lunatic Asylum, near Nottingham*, now comes under review. This institution is not a public or county establishment for the insane, strictly speaking, since it is governed by a committee appointed from among the voluntary subscribers and county justices; whilst first and second class patients are received, besides paupers belonging to the county. According to the medical officers' report, signed by Dr. Williams, the visiting physician, and Mr. Alderson, the superintendent, it appears that—

"On the 1st of January, 1852, 236 patients remained in the house; 123 males, 113 females. Since that period, 39 have been admitted; 19 males, 21 females: making a total of 275 cases under treatment during the year: viz., 140 males, and 134 females.

"Their general health has been uniformly good, although a few cases of diarrhœa, dysentery, and fever, occurred during the autumn, when those diseases prevailed extensively in the adjacent district; one case of fever assumed a typhoid character, and there were two deaths from diarrhœa and dysentery in chronic aged cases. All applications for the admission of private patients from the county (twelve in number) were received—viz., seven males and five females; of whom six are discharged recovered, one removed by his friends improved, one dead, and four remain; three of whom present many favourable symptoms of ultimate recovery; the remaining one is a man eighty years of age, in a state of senile imbecility.

"We are induced to name these cases at length, to show the proportion which may recover when the disease is early placed under judicious treatment: two-thirds were admitted into the asylum within six months after the attack was first perceived; and 50 per cent. have at present been discharged recovered, and are able to continue their ordinary occupation.

"From insufficient accommodation the whole of the parish patients could not be admitted; eleven males and sixteen females have been received; four of the males appeared likely to recover, seven were decidedly incurable, two the subjects of general paralysis, two of epilepsy, and two senile imbecility, aged respectively sixty-eight and seventy-two—the remaining one has been several years insane, and subject to violent paroxysms when at large. Eleven of the sixteen females presented indications of recovery; of whom five have been discharged cured, two are convalescent, three gradually improving, five are apparently incurable, and one has died. The daily average number of patients has been 240·49, thirty-seven have been discharged—viz., nine males, eleven females, cured; two males, one female, removed by friends; nine males, five females, died, giving a per centage of 4·91 deaths on the number treated. Their average age presents rather a high scale, the males being 57·5 years; females 53·3 years; as indicated in table 5. Most of the cases had been long resident in the institution, varying from three months to thirty-three years."

The same official authorities likewise remark—

"As in previous years, we have to record many of the cases as having been sent in a greatly reduced state of bodily health; also a large proportion of suicidal patients, two males and eleven females, exactly one-third of the whole admitted; several of these have attempted self-destruction, and others expressed themselves in such terms as to give to their friends the greatest unhappiness and fear. All such cases increase the responsibilities and anxieties of your officers, and add to their duties, more especially as we have no regular night attendants."

Respecting a very hopeless class of patients, namely, those labouring under epilepsy, and the treatment adopted for their relief, it is stated that the—

"Number of epileptics continue at a high scale—eleven per cent. This class is not free from danger to themselves and others, and till lately, medicine has had no very decided effect in relieving this malady. We have been induced, during the past year, to administer a new remedy—called Sambul, which was first introduced to our notice by a friend of high reputation, Dr. Boyd, of the Somerset County Asylum, where it had been used with apparent advantage.

"Accordingly, we selected two cases that appeared to us the most likely to receive benefit; in one of these the fits have much diminished in strength and frequency; the other has for some months been entirely free from epilepsy, and is now discharged. Shortly after its use, the fits gradually became less severe, and in six months ceased; he remained in the institution seven months after the last, and was discharged a month ago quite well. Up to the present time he has had no recurrence of the disease, and his mental and bodily health continue good. Several other patients have also taken the remedy, but without any very marked effect."

Before taking leave of the Nottingham Institution, we trust the medical officers will speedily carry out the intention they have expressed in the concluding paragraph of their recent report, in which my lords and gentlemen composing the Committee of Visitors are informed—

"That the reports cannot be so complete as we could wish, in consequence of the limited space allotted in the present form of annual report; we therefore request to be allowed in future, to print them in the form of a pamphlet, which will enable us to make the statistical tables more comprehensive and more easy of reference."

This is a laudable resolve, and will compensate for the limited nature of the present document we have now introduced to the acquaintance of our readers.

The next public institution for the insane we would bring under notice is the *Lunatic Asylum at Rainhill, in Lancashire*, of which the second annual report, by Mr. Eccleston, the surgeon-superintendent, lies on our table. At this establishment, 248 new patients were admitted during last year, the two sexes being equally divided—viz. 124 of each; 31 males and 49 females were discharged, recovered; whilst 31 male and 25 female lunatics died, which made a total mortality of 56; and gives a ratio of 22·59 per cent. of deaths, if compared with the number of admissions; the recoveries being 32·25 per cent. similarly calculated; besides which, 5 insane patients escaped.

An interesting table, containing the assigned cause of insanity, the form the disease assumed, and its duration in each case, with the special malady which apparently produced death, is likewise recorded. From this obituary, it appears drunkenness was the assigned cause in 7 of the fatal cases, and poverty or starvation in 4; but in many the facts being unknown, that column is consequently very imperfect. Respecting the form of insanity, and the disease apparently producing a fatal termination, the table is more minute. For instance, with reference to the types, 20 laboured under mania, and 9 manifested the acute form of that malady; whilst 12 patients died from general paralysis, 11 from apoplexy, and 10 from phthisis.

When speaking in a subsequent page, in regard to the management of lunatics, and superintendence of asylums for the insane, Mr. Eccleston observes, that the two great causes producing mental disease in patients admitted at the Rainhill Institution were *drunkenness and perverted ideas on religion*. Having said so generally, and even underlined the above words, so as to express a more decided opinion on the deleterious effects of such potent influences, he further says:—

"The symptoms of insanity induced by the abuse of alcoholic liquors are so well marked in their progress, and their termination is so certain and well-defined, as, in my opinion, to entitle them to careful and particular consideration.

"Alcoholismus chronicus, a disease frequently met with in our asylums, is characterised by tremulousness of the hands and arms, feebleness of the lower extremities, and a faltering utterance, in which last symptom, and in other respects also, it bears a close resemblance to the prodromic indications of general paralysis, especially to that form of the disease which admits of all the movements of the body, but where these movements are imperfect, inharmonious, and feeble. It differs, however, from general paralysis, in being, under favourable circumstances, curable. In the prodromic-paralytic form, when recent, and not extensively developed, and where it is limited to the nervous system alone without any organic lesion, we may generally assume the prognosis to be favourable; and, by judicious management, a tardy but complete restoration to the healthy state is the result. The duration of the malady is uncertain, and depends much upon the complications which generally attend it. In its progress, however, it is slow, but certain. We seldom see an unexpected or acute attack; and if sometimes we observe an apparently sudden development and rapid fatal termination, we may generally conclude that, from the insidious nature of the disease, we have allowed the first stages to pass by unnoticed.

"This disease is often observed in the patients of this asylum, and has frequently, in its latter stages, been confounded with general paralysis, until a perusal of the opinion of Dr. Huss turned attention to the subject, and gave rise to a careful and accurate observation of the phenomena peculiar to it."

Regarding the other point mooted, Mr. Eccleston considers—

"The cause of mental disease which ranks only second in potency and frequency to drunkenness, is perverted ideas on religion.

"I desire to guard myself from being understood to express any opinion, either favourable or adverse, to the theological doctrines of the Calvinists; I venture to express no such opinion, but limit myself strictly to the medical question of the influence these peculiar doctrines exert in the production of insanity. According to my own experience, all cases deserving the name of religious insanity are, with rare exceptions, the result of the Calvinistic theology. This opinion is fully supported by many authors of deservedly high reputation, and has recently a strong confirmation from the statistical tables of Hübertz, on the condition of the insane in the kingdom of Denmark, published in the 'Annales Médico-Psychologiques.' Dr. Hübertz shows that in every 1000 of the general population, which is Lutheran, only 2·10 are insane; while in every 1000 professing the Calvinistic creed, no less than 9·16 are insane. The wards of this institution have contained examples, far too numerous, of mind shipwrecked on the rocks of religious fatalism."

Allusion having been made to the places of nativity from whence lunatics were admitted during the past year, it is stated by the reporter that immigration entailed a considerable expense upon the county ratepayers, so large a proportion as one-third of the total admissions having been natives of other countries; whilst—

"The amount of the Irish element in the population of Liverpool will account for the large number of Roman Catholics in this asylum. The members of this Church form nearly one-fourth of the whole number of our inmates, and are nearly one-half as numerous as the inmates belonging to the Established Church."

The above fact is curious; and, seeing 14 inmates received during 1852 were from Wales, and 14 from Scotland, it is not only fair, but interesting, that attention should be directed to a matter which shows the liberality and benevolence exercised by the authorities of this institution.

According to the official Report of the Glasgow Royal Asylum, for 1852, it appears that—

"In the first quarter there were 64 patients admitted, in the second 69, in the third 76, and in the fourth only 57, making a total of 266, being 7 more than in 1851. Of these, 141 were males and 125 were females. As usual, the number of males admitted was greater than that of females. The increase of admissions has been in the male division of the West House, or in the higher class of patients. In this department the number of admissions was 40, while in 1851 it was 31. The total number of admissions into the East House was 197, and into the West House 69, showing a ratio of the higher to the lower class of patients of more than 1 to 3. By far the largest number of cases have been those coming under the general denomination of mania, under which term are included all those whose principal characteristic is excitement, without reference to the kind or number of delusions, as contra-distinguished from those whose general feature is depression, with or without delusions, or cases of melancholia; while of the former there were 164, there were of the latter only 63. The ratio of melancholia to mania is much higher in females than in males, there being 39 cases of melancholia to 75 of mania, or about one-half in females, and 24 to 89, or less than one-third in males. The unmarried are considerably greater in number than both the married and widowed. The number of unmarried males is greater than any other of this class. The ages of those admitted range between 15 and 75. The greater number are between the ages of 30 and 50; the number under 20 and above 50 being comparatively small."

Respecting the exciting causes of mental disease, the physician-superintendent, Dr. Mackintosh, by whom this valuable public document is drawn up, observes that intemperance, from the use of alcoholic agents, included the largest number of cases, there being 34 males and 22 females, or 56 instances of the above description. Such statements are very sad, and show how common drunkenness must prevail amongst the population resident in the west of Scotland. When adverting to these numerous examples of insanity recently admitted into the Glasgow Asylum, the Report says—

"They consist for the most part of cases which have been classified under the title of 'Oinomania.' Their general characteristic is an uncontrollable desire for ardent spirits; this desire is either constant, or merely felt at intervals; it may be of long standing, or of recent origin; so strong is it, that no claims of affection, no sense of duty, of interest, of honour, or of religion, can bar its progress. In cases of long standing, which are the most numerous, it is ineradicable or nearly so.

In those which are recent, it may be wholly recovered from. The danger of relapse, even in cases which have continued well for years, is great; many cases of this class being re-admissions. While some have numerous delusions and a confirmed state of mania, there are others who have so few or even no delusions, that in a short time, with the strongest asseverations of their complete recovery, they demand and obtain their liberation. At present it is impossible, without the concurrence of the patient, to confine him for a sufficient length of time to establish a cure, and the result is, that with liberty and opportunity, the same course is run as before, and the individual is again confined to be soon again liberated, and so on in endless alternation, to the ruin of all hope of recovery. There is no doubt that these unfortunate persons are diseased, and that their morbid state of body gives rise to that most insatiable craving for strong liquor which forces them, even against their own judgment, to do that which is wrong."

With reference to other exciting causes, we are told two persons became mad through political excitement, in consequence of the late general election; 11 were puerperal cases, a large number were included under the head of previous insanity: mental emotions, love, anxiety, grief, jealousy, over-study, and loss of property, were likewise ascertained to have acted as exciting causes; whilst epilepsy was similarly reported in 6 cases, and general paralysis in 5. Besides these influences, which often powerfully affect the human mind, and so produce insanity.

"The last class of cases which deserve notice are those included under the head of religious excitement. There are ten cases ascribed to this cause. It is far more likely, notwithstanding, that excitement on religious subjects was the consequence of the disease than the cause of it. The patient gradually becomes insane; his thoughts run on sacred subjects; he gives utterance to them, and immediately the cause is put down as religion, when, in fact, religion may have had nothing whatever to do with it as a cause. Although erroneous views of religion may in some cases lead to insanity, it is wrong to suppose that religion is in itself calculated to produce in a healthy mind any mental disorder, and there is nothing in the statistics of insanity to countenance or support this idea."

During the past year, 128 patients were discharged cured, and 50 died in this asylum. Of those recovered, 73 were males and 55 females: 88 being cases of mania, 39 of melancholia, and 1 of dementia. Again, of the 50 deaths reported, 31 were male, and 19 female patients; amongst whom 25 were cases of dementia, 17 of mania, and 8 of melancholia; 10 died from general paralysis, 8 by diarrhœa, 7 by phthisis, and 4 from epilepsy, the remaining 21 being by apoplexy, pneumonia, erysipelas, and other diseases.

In concluding his report, Dr. Mackintosh enters largely into the

Q Q 2

subject of treatment; and as that question, after all, is the most important that could occupy the attention of medical psychologists, we are therefore led to quote the following remarks before taking leave of this institution:—

"In regard to the treatment of diseases peculiar to the insane, the usual methods have been adopted. Depletion has rarely been had recourse to. The local detraction of blood has been occasionally found necessary and beneficial. A case seldom occurs here in which general bleeding could be practised with benefit. When the maniacal excitement at the first outset of the disease, even in the young and vigorous, is very great, the pulse may be full and bounding; and if, as unfortunately sometimes happens, previous to admission, large quantities of blood are abstracted, the consequence is, that the recovery of the patient is much retarded, if not altogether rendered hopeless. The prolonged use of stimulants and the most nourishing diet are imperatively required. The use of sedatives, both in calming the excitement of the maniacal, and relieving the misery of the melancholic, has been found to be of great service. Counter-irritation, by means of setons and blisters, applied to the neck or head, is constantly had recourse to, and in many cases with great benefit.

"With respect to the moral and other treatment, it has been nearly the same as has been hitherto practised in this institution. In the earlier stages of the disease, rest, seclusion, and the removal of every external source of excitement, are for the most part found necessary; in the later stages, when convalescence has begun, or the acute stage of the malady has passed over, employment both of body and mind is had recourse to, without which, neither mental nor bodily health can be maintained or improved. Walking, both within and without the grounds of the asylum; driving in the carriage and omnibus; agricultural and domestic labour; picking oakum; sewing, knitting; amusements of various kinds, such as the magic lantern and photography; social meetings, enlivened with music, both vocal and instrumental, have all been called into operation. The newspapers and periodicals of the day, both literary, scientific, and religious, and the newest publications —an ample supply of which is provided for the use of the patients—all tend to ameliorate their condition, and promote their recovery."

The next establishment to which we would direct attention, is the Crichton Royal Institution, near Dumfries, under the able superintendence of Dr. Browne. At this asylum, 135 patients, comprising 72 males and 63 females, were admitted during the year ending last November; 24 male lunatics, and 26 females, or a total of 50 individuals having recovered; whilst 25 died, of whom 11 were male, and 14 female inmates; the number remaining, when the report was presented, being 253 residents. Amongst the new patients, 89 were unmarried, 39 married, and 7 widowed; whilst 63, or nearly one-half, were from 30 to 50 years of age—that is, in the prime of life.

Respecting the type of insanity manifested, cases of mania were most numerous, 32 examples of that kind being reported amongst the new admissions; 20 were affected with melancholia, 20 exhibited fatuity, 14 laboured under mania with delusion, and 28 manifested different forms of monomania, besides examples of other varieties. In reference to the causes which produced mental disease in the patients recently admitted, 12 were ascribed to intemperance, 8 to disappointment, 7 to uterine irritation, 6 to masturbation, 6 to fever, 4 to puerperal condition, 3 to lactation, 3 to paralysis, and 3 to fear; besides special influences, such as religious impressions, over-study, or family affliction.

Like all the annual reports previously emanating from the pen of Dr. Browne, the present, which constitutes the thirteenth, will amply repay perusal. To give any analysis of its contents, either satisfactory to ourselves, or beneficial to our readers, is now impossible, consistent with the limited space at our command; consequently, we can only enumerate the subjects discussed by so able an author, leaving to others who may desire further information, to consult the original document, which extends to 36 closely-printed pages, and hence very different in that, as in other respects, from similar official statements sometimes issuing elsewhere.

After noticing the general results observed during the past year, as also the admissions, Dr. Browne alludes to the physical condition of the patients under treatment. The mortality recorded next occupies attention; then suicide, afterwards abstinence, and compulsory alimentation. Hallucination of senses, disorders of appetite, the mental condition of patients, and of their muscular system, are subsequently discussed. Convulsed patients, as also simulated convulsions, gyrators, dancers, climbers, grovellers, sedentary patients, hiders, attidudinizers, and rubbers, are afterwards mentioned, *seriatim;* then insensibility, laughers, tremblers, and violent patients occupy attention; lastly, amusements, social meetings, music, excursions, and even theatrical representations, are stated to have been brought into play, in order to improve and ameliorate the condition of those afflicted with that severest of human disorders—mental alienation. Each of these highly important questions having been amply investigated, Dr. Browne concludes his able report in the following paragraph:—

"There may be a dispute as to what is humane: there can be and there is no dispute that benevolence must be the groundwork of all future operations, or that to render such a principle effective it must be guided by science as well as by enlightened discrimination."

We next proceed to consider the *Second Annual Report of the County Lunatic Asylum, at Colney Hatch,* for 1853. It does not contain much extractable matter. The subjoined passages will convey to our readers a notion of the current statistics of the asylum.

"The Report presented to the court in January last stated the number of patients to be 383 males and 621 females. Your committee, finding that there were many of the lunatic poor remaining in private houses, proceeded to make arrangements for the reception of an additional number, and there are now 515 males and 729 females in the asylum.

"The total number admitted during the year has been 354 males and 270 females, of whom 42 males and 26 females have been discharged cured, 24 males and 17 females have been removed by parishes or friends, 53 males and 22 females have died, and 235 males and 205 females remain in the asylum. Most of those discharged cured were recent cases, and they left the asylum within six months from their admission. The great majority of the inmates being chronic cases, the number of those who are expected to recover is but small. The total number of deaths during the year have amounted to 119 males and 70 females out of the whole number of patients; but when it is stated that 68 did not survive three months from their admission, and that a large majority of those brought to the asylum since its opening have been long afflicted, and confined in private asylums, or workhouses, the number is probably not greater than might have been expected."

It appears that the weekly cost for maintenance per patient has been, for the last six months preceding the publication of the Report, 8*s.* 2*d.* On the subject of employment it is observed:—

"Seventy-five males are weekly employed on the farm and garden, and 144 more in other ways; while of the females 62 are engaged in the laundry, 6 in the kitchen, 96 as helpers in cleaning the wards and corridors, 2 at the officers' residences, 151 in needlework, and 11 in fancy-work."

In reference to the pathology of insanity, Mr. Tyerman, the resident medical officer on the male side of the establishment, makes the following sensible remarks:—

"The study of the pathology of the disease has been actively promoted during the year, and the constant existence of more or less extensive organic changes in the encephalon and other important viscera has been disclosed. In numerous cases these lesions were so remarkable as to excite surprise that life should have been so long continued; these facts bearing out the physiological observation that, whereas a sudden affection of comparatively small amount destroys at once all its functions, the brain is tolerant of great changes of a chronic nature. Such extensive changes were especially found in those cases of insidious origin associated with general paralysis, with which affection, however, various appearances are compatible. Sometimes great atrophy of the entire organ prevails, whilst in other instances, the affection appears attributable to altered capillary nutrition, the exact nature of the changes have yet to be discovered by nice chemical analysis, or perhaps the keen eye of the microscope. Although this peculiar form of disease alluded to under the term 'general paralysis,' does really eventually terminate in a complete paralysis of the muscles of animal life, powerful muscular movements (although ill-regulated) are compatible with its existence, and indeed not unfrequently an unnatural development of strength precedes the fatal issue. Notwithstanding the certainly fatal tendency of the disease by encroachment upon the cerebral functions, food is freely taken (often with voracity) and assimilated, the digestive organs performing their ordinary functions. The patient sometimes continues corpulent up to the period of his death, which occasionally appears attributable (as observed by others) to paralysis of the muscles (whose action is termed *reflex)* concerned in respiration."

With the view of affording additional amusement and occupation for the patients, an "exercising hall" has been erected. Mr. Tyerman, when referring to this fact, thus graphically describes the commendable effort of the official staff to bring the unhappy inmates of Colney Hatch within the sphere of cheerful moral influences. He says,—

"To this end the large exercising hall has not a little contributed, some active and meritorious ward attendants having occasionally marshalled in order of marching, and other military movements, 150 men all interested and hilarious at beat of drum and the lively music of the fife."

Our readers will have little difficulty in realizing the sublime spectacle of 150 lunatics "*marshalled in order of marching and other military movements, all interested and hilarious at beat of drum and the lively music of the fife.*" How such a scene would have gladdened the noble heart of Pinel, and have elevated the philanthropy of a Howard! Ought we not to consider the heroic feat thus classically described by Mr. Tyerman as the *chef-d'œuvre* in the moral treatment of the insane?

During a recent visit to the brilliant military encampment at Chobham, and whilst witnessing the wonderful and extraordinary evolutions of some of England's best and bravest soldiers, our imagination almost instinctively recurred to the far more ennobling and, when morally considered, transcendentally grander scene which Mr. Tyerman has so ably sketched in his last official report. Such an epoch in the treatment of the insane should be immortalized on canvass; and we hope, acting upon this suggestion, the Colney Hatch Committee of Management will lose no time in conferring with an accomplished British artist, and commission him to paint, for next annual exhibition of the Royal Academy, a sketch of the "*marching and other military movements*" of the 150 Colney Hatch lunatics, with their humane physician at their head, "*all*

*interested and hilarious*," not omitting to convey to the spectator some conception of the thrilling emotions and curative effects likely to be engendered and result from the "beat of drum and the lively music of the fife."

The *Annual Report of the Medical Officers of the Surrey Lunatic Asylum*, for 1853, presents many gratifying features. It contains a series of valuable tables, evidently drawn up with great care, relative to the condition of the patients when admitted, the character of the malady, &c. These tables we recommend to the patient study of all psychological physicians.

"At the date of our last report there were 853 patients in the asylum; since which, 360 have been admitted, and 329 have been discharged, or died; leaving, at the close of the year, 884.

"The total number of patients in the asylum, during the year, was 1213; the highest number at any time was 895, the lowest was 853; and the average number under treatment, during the whole period, was 868.

1852.

*Surrey County Lunatic Asylum.*

| | | | | M. | F. | Total. |
|---|---|---|---|---|---|---|
| Remaining 31st December, 1851 | | | | 374 | 479 | 853 |
| Admitted in 1852 | | | | 171 | 189 | 360 |
| Of whom have been discharged— | | | | 545 | 668 | 1213 |
| | M. | F. | Total. | | | |
| Recovered | 64 | 112 | 176 | | | |
| Removed not recovered | 28 | 24 | 52 | | | |
| Died | 57 | 44 | 101 | | | |
| | | | | 149 | 180 | 329 |
| Remaining in Asylum, 31st December, 1852 | | | | 396 | 488 | 884 |

"The number of recoveries is greater, and that of deaths is less, than in the preceding year, being nearly 14½ per cent. of the former, and little more than 8 per cent. of the latter. Seeing that this asylum, unlike the hospitals of Bethlem and St. Luke's, is obliged to receive every description of patients—paralytic, epileptic, idiotic, and dying, this result is very gratifying."

